# Comparing Public Perceptions and Preventive Behaviors During the Early Phase of the COVID-19 Pandemic in Hong Kong and the United Kingdom: Cross-sectional Survey Study

**DOI:** 10.2196/23231

**Published:** 2021-03-08

**Authors:** Leigh Bowman, Kin On Kwok, Rozlyn Redd, Yuanyuan Yi, Helen Ward, Wan In Wei, Christina Atchison, Samuel Yeung-Shan Wong

**Affiliations:** 1 MRC Centre for Global Infectious Disease Analysis and Abdul Latif Jameel Institute for Disease and Emergency Analytics (J-IDEA) School of Public Health Imperial College London United Kingdom; 2 JC School of Public Health and Primary Care The Chinese University of Hong Kong Hong Kong Hong Kong; 3 Stanley Ho Centre for Emerging Infectious Diseases The Chinese University of Hong Kong Hong Kong Hong Kong; 4 Shenzhen Research Institute The Chinese University of Hong Kong Hong Kong Hong Kong; 5 Patient Experience Research Centre School of Public Health Imperial College London London United Kingdom

**Keywords:** COVID-19, novel coronavirus, pandemic, behavioural response, risk perceptions, anxiety, comparative, Hong Kong, United Kingdom

## Abstract

**Background:**

Given the public health responses to previous respiratory disease pandemics, and in the absence of treatments and vaccines, the mitigation of the COVID-19 pandemic relies on population engagement in nonpharmaceutical interventions. This engagement is largely driven by risk perception, anxiety levels, and knowledge, as well as by historical exposure to disease outbreaks, government responses, and cultural factors.

**Objective:**

The aim of this study is to compare psychobehavioral responses in Hong Kong and the United Kingdom during the early phase of the COVID-19 pandemic.

**Methods:**

Comparable cross-sectional surveys were administered to adults in Hong Kong and the United Kingdom during the early phase of the epidemic in each setting. Explanatory variables included demographics, risk perception, knowledge of COVID-19, anxiety level, and preventive behaviors. Responses were weighted according to census data. Logistic regression models, including effect modification to quantify setting differences, were used to assess the association between the explanatory variables and the adoption of social distancing measures.

**Results:**

Data from 3431 complete responses (Hong Kong, 1663; United Kingdom, 1768) were analyzed. Perceived severity of symptoms differed by setting, with weighted percentages of 96.8% for Hong Kong (1621/1663) and 19.9% for the United Kingdom (366/1768). A large proportion of respondents were abnormally or borderline anxious (Hong Kong: 1077/1603, 60.0%; United Kingdom: 812/1768, 46.5%) and regarded direct contact with infected individuals as the transmission route of COVID-19 (Hong Kong: 94.0%-98.5%; United Kingdom: 69.2%-93.5%; all percentages weighted), with Hong Kong identifying additional routes. Hong Kong reported high levels of adoption of various social distancing measures (Hong Kong: 32.6%-93.7%; United Kingdom: 17.6%-59.0%) and mask-wearing (Hong Kong: 98.8% (1647/1663); United Kingdom: 3.1% (53/1768)). The impact of perceived severity of symptoms and perceived ease of transmission of COVID-19 on the adoption of social distancing measures varied by setting. In Hong Kong, these factors had no impact, whereas in the United Kingdom, those who perceived their symptom severity as “high” were more likely to adopt social distancing (adjusted odds ratios [aORs] 1.58-3.01), and those who perceived transmission as “easy” were prone to adopt both general social distancing (aOR 2.00, 95% CI 1.57-2.55) and contact avoidance (aOR 1.80, 95% CI 1.41-2.30). The impact of anxiety on adopting social distancing did not vary by setting.

**Conclusions:**

Our results suggest that health officials should ascertain baseline levels of risk perception and knowledge in populations, as well as prior sensitization to infectious disease outbreaks, during the development of mitigation strategies. Risk should be communicated through suitable media channels—and trust should be maintained—while early intervention remains the cornerstone of effective outbreak response.

## Introduction

In December 2019, a novel coronavirus, SARS-CoV-2, emerged in Wuhan, Hubei Province, China, and spread rapidly worldwide, forming the second pandemic of the 21st century [1]. The progression of the disease has varied by region. As of August 2, 2020, there have been at least 17 million cases of COVID-19 and over 670,000 deaths globally [2].

Prior to the availability of effective treatments and vaccines, strategies to mitigate the impact of the pandemic have been primarily nonpharmaceutical [3], mainly focusing on public health promotion of using simple but effective preventive measures [4,5]. Many important control strategies currently promoted by governments require public participation, either through direct adoption of preventative behaviors, such as handwashing or wearing face masks, or through compliance with social distancing policies, such as recommendations to avoid public transport and mass gatherings.

Previous studies of the severe acute respiratory syndrome and influenza pandemics showed that governments should account for risk perception and anxiety when promoting preventative measures. There is evidence that higher perceived risk of infection is associated with increased adoption of precautionary measures [6,7], while increased anxiety has also been shown to increase the likelihood that people will engage in protective behaviors [8]. Moreover, longitudinal data suggest that these perceptions, behaviors and anxieties change with context and over time as uncertainty about disease severity decreases and knowledge of transmission increases [9].

During the current COVID-19 pandemic, researchers have examined public risk perceptions and knowledge in various countries, including Finland [10], Israel [11], Italy [12], Nigeria [13], the United States [14,15], South Korea [16] and Vietnam [17]. However, only a few studies have identified the factors associated with greater adoption of preventative measures or how these associations vary by context. In Hong Kong, both greater understanding of COVID-19 and increased anxiety were associated with greater adoption of social distancing behaviors [5], whereas in the United Kingdom, there was a significant socioeconomic gradient in the ability to adopt and comply with social distancing measures, specifically the ability to work from home and the ability to self-isolate [18].

This initial evidence that there is variation across context in affective responses, risk perceptions, and the impact of sociodemographic factors on the uptake of preventative behaviors has significant implications when tailoring policies. To elucidate these relationships, a more thorough comparative analysis is required. However, studies in different countries often use different metrics to measure the same behavior, which can lead to difficulty when interpreting the significance of heterogeneous contexts.

In this study, we examined and compared public perception and adoption of preventive behaviors in response to the early phase of the COVID-19 pandemic in two different settings: Hong Kong and the United Kingdom. We further investigated the factors associated with greater adoption of different types of social distancing measures. Our results have immediate implications on how health officials plan and communicate strategies to mitigate the ongoing COVID-19 pandemic to communities.

## Methods

### Study Design and Recruitment

In Hong Kong and the United Kingdom, cross-sectional surveys were conducted during the early phase of the COVID-19 pandemic, when limited government-level interventions were in place [5,18]. The survey period in Hong Kong was from January 24 to February 13, 2020, and that in the United Kingdom was from March 17 to 18, 2020. In Hong Kong, the first laboratory-confirmed case of COVID-19 was reported on January 23, 2020, and the number of cases rose to 53 by February 13, 2020 [19]; meanwhile, in the United Kingdom, the first two laboratory-confirmed cases were reported on January 31, 2020, and the number of cases rose to 2626 by March 18, 2020 [20].

In Hong Kong, all 452 district councilors were invited to distribute an open web-based survey by sharing a survey link and promotion messages on their webpages, social media platforms, or any channels which they usually used to convey information to their targeted residents. Individuals aged ≥18 years who understood Chinese and lived in Hong Kong were eligible to participate [5]. Respondents were compensated with HK $10 (US $1.29) in the form of a cash coupon. In the United Kingdom, the web-based survey was not open (users were required to log in) and was administered by YouGov, a market research company, to members of its panel of ≥800,000 individuals (aged ≥18 years) as part of their omnibus survey [21]. Our UK sample was obtained through a nonprobabilistic active sampling method, and emails were sent to randomly selected individuals with particular characteristics to match the proportions of people with those characteristics in the 2011 UK census. No incentive was involved in the UK survey. More details of the survey design are described elsewhere [5,18].

### Study Instruments

The study instruments are freely available on the web (Hong Kong: [22]; United Kingdom: [23]). The UK questionnaire was adapted from the Hong Kong version, with feedback from 20 members of the public (of different backgrounds) to improve its relevance and usability in the UK context. This process led to some discrepancies in the questions or answer choices; however, the two questionnaires were largely similar.

Sociodemographic variables included age, sex, educational attainment, and employment status. Anxiety level was measured using the Hospital Anxiety and Depression scale–Anxiety (HASD-A) (0-7=normal; 8-10=borderline abnormal; 11-21=abnormal) [24]. Risk perception toward COVID-19 was measured by perceived severity of symptoms if the respondent was infected with COVID-19 (Hong Kong, question 35; United Kingdom, question GIC_Q29). Knowledge of COVID-19 was assessed by asking whether COVID-19 could be transmitted through various routes (Hong Kong, question 45; United Kingdom, question GIC_Q33), including direct human exposure (eg, physical contact or a face-to-face conversation with someone who is infected with SARS-CoV-2 with or without symptoms) and other types of exposure (eg, visiting wet markets or consumption of wild animal meat). From knowledge of COVID-19, perceived ease of transmission was regarded as “easy” if the virus was deemed to be transmitted through face-to face conversation with asymptomatic infectees and as “difficult” otherwise. Respondents were also asked about the sources from which they retrieved information about COVID-19 and their perceived reliability of these sources (Hong Kong, questions 40 and 43; United Kingdom, questions GIC_Q30 and GIC_Q32). In addition, they were asked about the adoption of preventative behaviors to prevent the transmission of COVID-19 (Hong Kong, question 46; United Kingdom, questions GIC_Q34a and GIC_Q34b). Three types of preventative measures were considered: personal hygiene, social distancing, and travel avoidance.

### Data Analysis

Descriptive statistics for all variables present the number of respondents and the raw or weighted percentages. In this manuscript, weighted percentages were used for description except for demographics. The responding samples were weighted to be representative of the United Kingdom (2011 census [25]) and Hong Kong (2016 by-census [26]) adult populations using the raking method [27]. Each data point was given a weight so that the marginal proportions of the demographics in the survey (age, sex, region, education level, and [United Kingdom only] social grade) were similar to those in the census. Chi-square goodness-of-fit tests were used for comparing characteristics across settings. Multivariate logistic regression models were used to identify sociodemographic and psychosocial factors associated with the adoption of three types of social distancing: (1) general measures, specified by avoiding crowded places, social events and going out; (2) contact measures, specified by avoiding contact with individuals who had fever or respiratory symptoms and who had been to affected areas recently; and (3) work measures, specified by avoiding going to work.

Common and comparable sociodemographic factors considered in separate analytical studies [5,18] were included in this comparative analysis. These factors were considered as confounders in the association between psychosocial factors (including anxiety level, perceived severity, and perceived ease of transmission) and adoption of social distancing measures. Further, these associations (between each aforementioned psychological factor and each type of social distancing measure) were considered a priori to be affected by setting. Therefore, we examined the effect modifications due to setting using interaction terms in the baseline models, which can be interpreted as the difference in the estimated effects of psychosocial factors on adopting social distancing measures due to different settings. Adjusted odds ratios (aORs) and 95% confidence intervals were estimated. Associations with *P*<.05 in the adjusted analyses were considered to be statistically significant. Analyses were conducted in R, version 3.6.3 (the R Project) and STATA, version 11 (StataCorp LLC).

### Ethical Approval

The study was approved by the Imperial College London Research Ethics Committee (reference number: 20IC5861) and the Survey and Behavioral Research Ethics Committee of The Chinese University of Hong Kong (reference number: SBRE-19-625).

## Results

### Survey Responses

In Hong Kong, there were initially 2478 clicks on the survey link. After removing 763 cases with missing demographics and 52 cases with ambiguous responses on the perceived ease of transmission, 1663 complete cases were included in the analysis. In the United Kingdom, 2500 individuals were approached, and the response rate was 84.3% (2108/2500). After excluding cases with missing demographics or perceived severity and cases with ambiguous responses on the perceived ease of transmission, 1768 cases were included in the analysis.

### Demographic Differences

There were significant differences in the sociodemographic characteristics of the study respondents between the two settings. Hong Kong respondents were younger, with 26.0% (433/1663) aged 18-24 years, compared with 9.4% (166/1768) for the United Kingdom (*P*<.001) (Table 1).

**Table 1:** Characteristics of the study respondents in the United Kingdom and Hong Kong (all *P* values <.001 as determined by chi-square goodness-of-fit test).

Characteristics		United Kingdom (n=1768)			Hong Kong (n=1663)			
		n	% (unweighted)	% (weighted)		n	% (unweighted)	% (weighted)
**Age (years)**								
	18-24	166	9.4	9.9		433	26.0	17.0
	25-34	243	13.7	14.3		535	32.2	23.5
	35-44	335	18.9	19.5		370	22.2	23.9
	45-54	300	17.0	17.7		193	11.6	22.2
	≥55	724	41.0	38.6		132	7.9	13.4
**Sex**								
	Female	936	52.9	51.8		1141	68.6	57.1
	Male	832	47.1	48.2		522	31.4	42.9
**Education attainment**								
	No formal qualification/lower secondary or below	100	5.7	5.5		53	3.2	9.9
	Secondary level qualification/higher secondary	738	41.7	43.2		292	17.6	32.5
	Postsecondary but below degree	334	18.9	18.3		267	16.1	16.2
	Degree or above	596	33.7	32.9		1051	63.2	41.5
**Employment status**								
	Employer/employee	1025	58.0	59.6		1135	68.3	66.0
	Full-time student	90	5.1	5.3		278	16.7	12.5
	Unemployed/not working	172	9.7	10.4		206	12.4	17.2
	Retired	481	27.2	24.7		44	2.6	4.3
**Perceived severity^a^**								
	Level 1	96	5.4	5.2		1071	64.4	65.0
	Level 2	270	15.3	14.7		550	33.1	31.8
	Level 3	1058	59.8	60.2		32	1.9	2.2
	Level 4	320	18.1	18.5		7	0.4	0.7
	Level 5	24	1.4	1.4		3	0.2	0.3
**Worry about COVID-19**								
	Very worried	536	30.3	30.1		852	51.2	49.4
	Fairly worried	858	48.5	48.4		723	43.5	43.2
	Neutral/don’t know	5	0.3	0.3		40	2.4	3.2
	Not very worried	295	16.7	16.9		1	0.1	0.1
	Not at all worried	74	4.2	4.3		47	2.8	4.1
**Anxiety level**								
	Normal	956	54.1	53.5		586	35.2	40.0
	Borderline abnormal	336	19.0	19.4		512	30.8	27.3
	Abnormal	476	26.9	27.1		565	34.0	32.7

^a^Level 1=very serious (Hong Kong)/life-threatening (United Kingdom); Level 2=serious (Hong Kong)/severe (eg, may need care and treatment in hospital) (United Kingdom); Level 3=neutral (Hong Kong)/moderate (eg, may need self-care and rest in bed) (United Kingdom); Level 4=not serious (Hong Kong)/mild (eg, can go about daily tasks normally) (United Kingdom); Level 5=not serious at all (Hong Kong)/no symptoms (United Kingdom).

The Hong Kong sample contained a greater proportion of women (Hong Kong: 1141/1663, 68.6%, vs United Kingdom: 936/1768, 52.9%; *P*<.001), and respondents educated to university degree level or above (Hong Kong: 1051/1663, 63.2%, vs United Kingdom: 596/1768, 33.7%; *P*<.001). Employment status reflected the age structure of the respondents in each setting, with a greater proportion of UK respondents in the retired category (Hong Kong: 44/1663, 2.6%, vs United Kingdom: 481/1768, 27.2%; *P*<.001) (Table 1).

### Perceptions and Beliefs

Higher perceived severity of COVID-19 was observed among Hong Kong respondents, with 96.8% (1621/1663) rating the symptoms of COVID-19 infection as serious or very serious compared with only 19.9% (366/1768) of the UK respondents. In terms of levels of concern, 92.6% (1575/1663) of the Hong Kong sample responded that they felt very or fairly worried, compared with 78.5% (1394/1768) of the UK sample. The HADS-A scores reflected similar trends, with 60.0% (1077/1663) of the Hong Kong sample recording an abnormal or borderline abnormal result, compared with 46.5% (812/1768) of the UK sample (Table 1).

### Knowledge and Information Sources

The majority of respondents regarded direct contact with infected individuals (Hong Kong: 94.0%-98.5%; United Kingdom: 69.2%-93.5%) or virus-contaminated environments (Hong Kong: 1594/1663, 96.3%; United Kingdom: 1411/1768, 79.5%) as the primary means of virus transmission (Table 2). However, respondents from Hong Kong identified a far broader scope of transmission routes. A much larger proportion of Hong Kong respondents regarded wild animal meat (Hong Kong: 1546/1663, 93.4%; United Kingdom: 199/1768, 11.3%), wet markets (Hong Kong: 1342/1663, 81.1%; United Kingdom: 374/1768, 21.5%), imported seafood (Hong Kong: 1199/1663, 70.9%; United Kingdom: 258/1768, 14.8%) and imported goods (Hong Kong: 1101/1663, 66.6%; United Kingdom: 209/1768, 12.1%) as potential exposure sources than their UK counterparts. There was also significant variation across use and reliability of information sources (Table S1 in Multimedia Appendix 1). The majority of respondents deemed health professionals to be reliable (>80% in both Hong Kong and the United Kingdom); however, few could access them (Hong Kong: 86/1663, 4.8%; United Kingdom: 202/1768, 11.5%). In addition, most UK respondents (1602/1768, 90.7%) considered official websites to be reliable, compared to 15.6% (260/1663) among Hong Kong respondents at the beginning of the pandemic.

**Table 2:** Knowledge of COVID-19 transmission.

“Are the following transmission routes of COVID-19?”		Respondents answering “yes”					
		United Kingdom (n=1768)			Hong Kong (n=1663)		
		n	% (unweighted)	% (weighted)	n	% (unweighted)	% (weighted)
**Contact**							
	Face-to-face conversation (no physical contact) with someone who has SARS-CoV-2 but no symptoms	1234	69.8	69.2	1564	94.0	94.0
	Face-to-face conversation (no physical contact) with someone who has SARS-CoV-2 with symptoms	1398	79.1	78.7	1616	97.2	96.8
	Physical contact with someone who has SARS-CoV-2 but no symptoms	1580	89.4	89.0	1635	98.3	98.1
	Physical contact with someone with SARS-CoV-2 who has symptoms	1657	93.7	93.5	1644	98.9	98.5
**Transmission mode**							
	Droplets	N/A^a^	N/A	N/A	1649	99.2	99.2
	Aerosol when infected people cough or sneeze	N/A	N/A	N/A	1478	88.9	91.2
	Being in close contact (ie, within 2 meters) with someone who has SARS-CoV-2 when they cough or sneeze	1604	90.7	90.4	N/A	N/A	N/A
	Being further away (ie, further than 2 meters) from someone who has SARS-CoV-2 when they cough or sneeze	615	34.8	34.8	N/A	N/A	N/A
**Others**							
	Contact with virus-contaminated environment	1411	79.8	79.5	1594	95.9	96.3
	Consumption of wild animal meat	199	11.3	11.3	1546	93.0	93.4
	Visiting a wet market	374	21.2	21.5	1342	80.7	81.1
	Consumption of seafood imported from specific regions^b^	258	14.6	14.8	1199	72.1	70.9
	Consumption/use of products imported from specific regions^b^	209	11.8	12.1	1101	66.2	66.6

^a^N/A: not applicable.

^b^Specific regions refer to China (United Kingdom)/Wuhan (Hong Kong).

### Adoption of Social Distancing Measures

**Figure 1:**
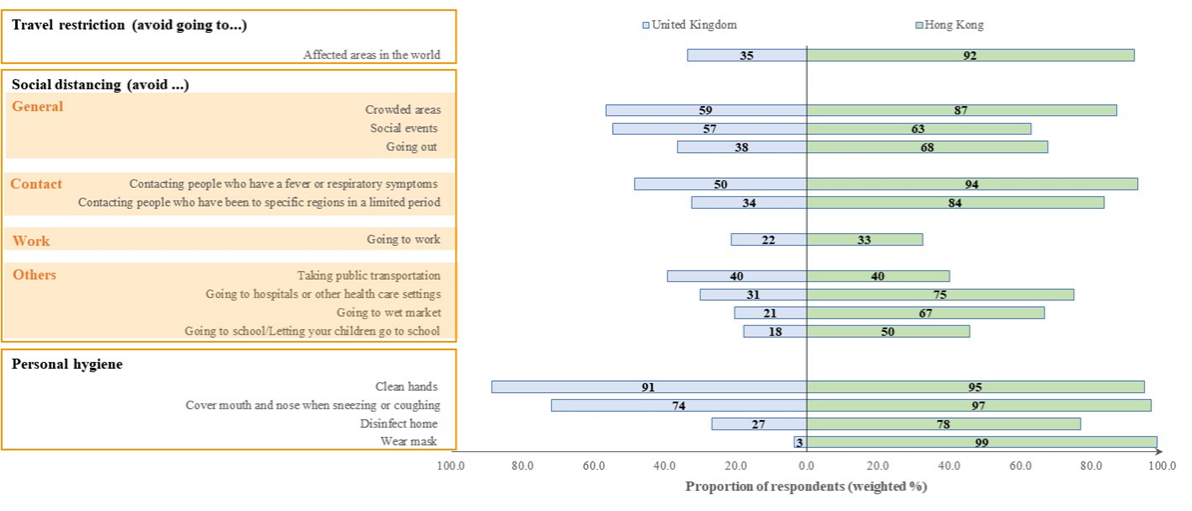
Adoption of precautionary measures against COVID-19. “Affected areas” refers to China (Hong Kong)/affected areas in the world (United Kingdom); “Specific regions in a limited period” refers to Wuhan in the past one month (Hong Kong)/affected areas in the past 14 days (United Kingdom). The “Going to work” category only included respondents who were employees or employers (n=2160), and the “Going to school/letting your children go to school” category only included respondents who were full-time students or had at least one child (n=1239).

There were variations in the weighted proportions of Hong Kong and the UK respondents who adopted precautionary measures against COVID-19 (Figure 1; Table S2 in Multimedia Appendix 1). Hong Kong respondents reported higher levels of adoption across all social distancing and personal hygiene measures. In particular, 98.8% (1647/1663) of Hong Kong respondents reported wearing a face mask, compared to 3.1% (53/1768) among the UK respondents. General measures were adopted by 63.1%-87.2% and 37.8%-59.0% of respondents in Hong Kong and the United Kingdom, respectively. Contact measures were adopted by 83.8%-93.7% and 33.7%-50.1% of respondents in Hong Kong and the United Kingdom, respectively. Work measures were reported by 32.6% (402/1135) and 22.5% (231/1025) of respondents in Hong Kong and the United Kingdom, respectively.

Sociodemographic factors were associated with the three social distancing measures (Table S3 in Multimedia Appendix 1; Table 3). The UK respondents were significantly less likely than their Hong Kong counterparts to adopt social distancing measures (Table S3 in Multimedia Appendix 1, OR 0.08-0.53, *P*<.001; Table 3, aOR 0.08-0.70, *P*<.001). When adjusting for differences between settings, general measures were less likely to be adopted by male respondents (aOR 0.82; 95% CI 0.71-0.95) but more likely to be adopted by the unemployed (aOR 1.65; 95% CI 1.30-2.09) or retired (aOR 1.92; 95% CI 1.43-2.59). Contact measures were less likely to be adopted by male respondents (aOR 0.74; 95% CI:0.63-0.88) but more likely to be adopted by those who were retired (aOR 1.40; 95% CI 1.03-1.91). Finally, work measures were less likely to be adopted by respondents aged ≥55 years (aOR 0.60; 95% CI 0.39-0.93).

**Table 3:** Factors associated with the adoption of different social distancing measures.

Factor		Types of social distancing measures													
		General^a^ (n=3431) (Model 1)				Contact^b^ (n=3431) (Model 2)					Work^c^ (n=2160) (Model 3)				
		aOR^d^ (95% CI)		*P* value		aOR (95% CI)		*P* value		aOR (95% CI)			*P* value		
**Age (years)**															
	18-24		Reference		N/A^e^		Reference		N/A			Reference			N/A
	25-34		1.54 (1.16-2.05)		.003		0.96 (0.68-1.35)		.81			1.00 (0.72-1.39)			.99
	35-44		1.25 (0.93-1.68)		.13		0.74 (0.53-1.05)		.09			0.95 (0.68-1.33)			.77
	45-54		1.30 (0.95-1.79)		.10		0.67 (0.47-0.97)		.03			0.72 (0.49-1.05)			.09
	55+		0.99 (0.70-1.41)		.97		0.81 (0.55-1.19)		.28			0.60 (0.39-0.93)			.02
**Sex**															
	Female		Reference		N/A		Reference		N/A			Reference			N/A
	Male		0.82 (0.71-0.95)		.01		0.74 (0.63-0.88)		<.001			0.95 (0.78-1.16)			.62
**Education attainment**															
	No formal qualification/lower secondary or below		Reference		N/A		Reference		N/A			Reference			N/A
	Secondary level qualification/higher secondary		0.99 (0.68-1.44)		.96		0.96 (0.64-1.44)		.85			1.04 (0.46-2.34)			.92
	Postsecondary but below degree		1.06 (0.72-1.55)		.78		1.12 (0.74-1.71)		.58			1.09 (0.48-2.47)			.83
	Degree or above		1.27 (0.87-1.83)		.21		0.98 (0.66-1.47)		.94			1.94 (0.88-4.28)			.10
**Employment status**															
	Employed		Reference		N/A		Reference		N/A			N/A			N/A
	Full-time student		1.35 (0.98-1.85)		.07		1.08 (0.73-1.59)		.70			N/A			N/A
	Unemployed		1.65 (1.30-2.09)		<.001		1.20 (0.91-1.58)		.20			N/A			N/A
	Retired		1.92 (1.43-2.59)		<.001		1.40 (1.03-1.91)		.03			N/A			N/A
**Setting**															
	Hong Kong		Reference		N/A		Reference		N/A			Reference			N/A
	United Kingdom		0.35 (0.30-0.41)		<.001		0.08 (0.07-0.10)		<.001			0.70 (0.57-0.87)			<.001

^a^General: avoiding going to crowded areas; going to social events; and going out.

^b^Contact: avoiding contacting individuals who had a fever or respiratory symptoms and had been to Wuhan in the past month (Hong Kong)/affected areas in the past 14 days (United Kingdom).

^c^Work: avoiding going to work.

^d^aOR: adjusted odds ratio.

^e^N/A: not applicable.

The impact of perceived severity of infection (Table 4) and perceived ease of transmission (Table 5) on the adoption of social distancing behaviors varied by setting. In Hong Kong, these factors had no impact, whereas in the United Kingdom, those who perceived COVID-19 infection as serious were more likely to adopt all social distancing measures (aOR 1.58-3.01), and those who perceived transmission of SARS-CoV-2 as easy were more likely to adopt both general social distancing measures (Model 4.2, aOR 2.00, 95% CI 1.57-2.55) and contact measures (Model 5.2, aOR 1.80, 95% CI 1.41-2.30). On the other hand, the impact of anxiety on the adoption of social distancing behaviors did not significantly differ by setting (Table 6). Those with borderline abnormal (Hong Kong, aOR 1.26-1.62; United Kingdom, aOR 1.36-1.48) or abnormal (Hong Kong, aOR:1.82-2.09; United Kingdom, aOR:1.40-2.40) HADS-A scores were more likely to adopt all three types of social distancing measures compared to those with normal anxiety levels.

**Table 4:** Setting-specific effects and effect modification (by setting) of perceived severity on the adoption of social distancing measures. The models have been adjusted for all covariates.

Settings and variables			Types of social distancing															
			Model 4.1						Model 5.1						Model 6.1			
			General^a^ (n=3431)						Contact^b^ (n=3431)						Work^c^ (n=2160)			
				aOR^d^ (95% CI)		*P* value			aOR (95% CI)			*P* value			aOR (95% CI)			*P* value
**Hong Kong**																		
	Not serious^e^			Reference		N/A^f^			Reference			N/A			Reference			N/A
	Serious^g^			0.93 (0.50-1.74)		.82			1.71 (0.85-3.47)			.13			0.63 (0.30-1.30)			.21
**United Kingdom**																		
	Not serious			Reference		N/A			Reference			N/A			Reference			N/A
	Serious			3.01 (2.35-3.86)		<.001			1.90 (1.48-2.43)			<.001			1.58 (1.06-2.37)			.03
Effect modification^h^			3.24 (1.65-6.35)		<.001			1.11 (0.52-2.34)			.79			2.52 (1.09-5.80)			.03	

^a^General: avoiding going to crowded areas and social events and going out.

^b^Contact: avoiding contacting individuals who had a fever or respiratory symptoms and had been to Wuhan in the past one month (Hong Kong) or affected areas in the past 14 days (United Kingdom).

^c^Work: avoiding going to work.

^d^aOR: adjusted odds ratio.

^e^For perceived severity, “not serious” refers to levels 3-5 (neutral to not serious at all, Hong Kong; moderate to no symptoms, United Kingdom).

^f^N/A: not applicable.

^g^For perceived severity, “serious” refers to levels 1-2 (very serious to serious, Hong Kong; life-threatening to severe, United Kingdom).

^h^Measures the difference of the effect being considered due to difference in setting; its value is the ratio of the two setting-specific effects.

**Table 5:** Setting-specific effects and effect modification (by setting) of perceived ease of transmission on the adoption of social distancing measures. The models have been adjusted for all covariates.

Settings and variables			Types of social distancing															
			Model 4.2						Model 5.2						Model 6.2			
			General^a^ (n=3431)						Contact^b^ (n=3431)						Work^c^ (n=2160)			
				aOR^d^ (95% CI)		*P* value			aOR (95% CI)			*P* value			aOR (95% CI)			*P* value
**Hong Kong**																		
	Difficult^e^			Reference		N/A^f^			Reference			N/A			Reference			N/A
	Easy^g^			1.15 (0.77-1.74)		.50			1.00 (0.58-1.71)			.99			0.61 (0.37-1.03)			.07
**United Kingdom**																		
	Difficult			Reference		N/A			Reference			N/A			Reference			N/A
	Easy			2.00 (1.57-2.55)		<.001			1.80 (1.41-2.30)			<.001			1.34 (0.96-1.87)			.09
Effect modification^h^			1.73 (1.07-2.79)		.02			1.81 (1.00-3.28)			.05			2.18 (1.18- 4.04)			.01	

^a^General: avoiding going to crowded areas and social events and going out.

^b^Contact: avoiding contacting individuals who had a fever or respiratory symptoms and had been to Wuhan in the past one month (Hong Kong) or affected areas in the past 14 days (United Kingdom).

^c^Work: avoiding going to work.

^d^aOR: adjusted odds ratio.

^e^For perceived ease of transmission, “difficult” means that the virus cannot be transmitted by face-to face conversation with someone who has SARS-CoV-2 but no symptoms.

^f^N/A: not applicable.

^g^For perceived ease of transmission, “easy” means that the virus can be transmitted by face-to face conversation with someone who has SARS-CoV-2 but no symptoms.

^h^Measures the difference of the effect being considered due to difference in setting; its value is the ratio of the two setting-specific effects.

**Table 6:** Setting-specific effects and effect modification (by setting) of anxiety level on the adoption of social distancing measures. The models have been adjusted for all covariates.

Settings and variables			Types of social distancing															
			Model 4.3						Model 5.3						Model 6.3			
			General^a^ (n=3431)						Contact^b^ (n=3431)						Work^c^ (n=2160)			
				aOR^d^ (95% CI)		*P* value			aOR (95% CI)			*P* value			aOR (95% CI)			*P* value
**Hong Kong**																		
	Normal			Reference		N/A^e^			Reference			N/A			Reference			N/A
	Borderline abnormal			1.62 (1.27-2.06)		<.001			1.26 (0.93-1.70)			.14			1.51 (1.10-2.06)			.01
	Abnormal			2.09 (1.64-2.66)		<.001			1.85 (1.34-2.56)			<.001			1.82 (1.34-2.48)			<.001
**United Kingdom**																		
	Normal			Reference		N/A			Reference			N/A			Reference			N/A
	Borderline abnormal			1.48 (1.11-1.96)		.01			1.36 (1.02-1.80)			.03			1.37 (0.92-2.02)			.12
	Abnormal			2.40 (1.87-3.09)		<.001			1.76 (1.37-2.27)			<.001			1.40 (0.99-1.98)			.06
Effect modification^f^ (borderline abnormal)			0.91 (0.63-1.33)		.64			1.08 (0.71, 1.63)			.71			0.91 (0.55-1.49)			.70	
Effect modification (abnormal)			1.15 (0.82-1.62)		.42			0.95 (0.63-1.42)			.80			0.77 (0.48-1.21)			.26	

^a^General: avoiding going to crowded areas and social events and going out.

^b^Contact: avoiding contacting individuals who had a fever or respiratory symptoms and had been to Wuhan in the past one month (Hong Kong) or affected areas in the past 14 days (United Kingdom).

^c^Work: avoiding going to work.

^d^aOR: adjusted odds ratio.

^e^N/A: not applicable.

^f^Measures the difference of the effect being considered due to difference in setting; its value is the ratio of the two setting-specific effects.

## Discussion

### Principal Results

This study compared the initial public perceptions and preventative behaviors during the COVID-19 pandemic across Hong Kong and the United Kingdom. The adoption of social-distancing measures was higher in Hong Kong than in the United Kingdom. Risk perception and knowledge of COVID-19 were consistently and significantly higher in Hong Kong; however, for the United Kingdom, respondents’ adoption of preventive behaviors was associated with two metrics: if transmission was considered to be “easy” and the perceived severity was “severe,” UK respondents were more likely to adopt preventive behaviors. Anxiety was a driver of behavior change in both settings: those who were more anxious were more likely to adopt preventative measures. This behavior is consistent with the wider literature surrounding the adoption of precautionary measures [6,7] and provides further evidence that anxiety drives protective behaviors, such as handwashing [8], an effective intervention against the transmission of respiratory diseases [28].

### Implications

This study has three implications. First, health officials should account for context-specific baseline levels of risk perception and knowledge when designing and promoting mitigation strategies. The evidence presented in this study demonstrates that geographical and sociocultural context is important in terms of both how people understand risk and how risk drives behavior. Although the social, historical, and cultural heterogeneity between Hong Kong and the United Kingdom likely contributes to the results of this study, the importance of intrinsic factors such as population sensitization via past infectious disease outbreaks and state-led health promotion campaigns should not be underestimated. In other studies, public perceptions of these factors have been found to be significant drivers of adopting preventative behaviors during previous epidemics [29], while conceptions of personal risk have been connected to individuals’ understanding of local disease prevalence and severity [30-32]. Therefore, assessment of the baseline population knowledge, attitudes, and practices (KAP) and subsequent continual monitoring throughout the pandemic are essential to effective context-specific pandemic preparedness plans.

Second, risk communication should build upon baseline KAP outcomes, and trust should be developed across suitable media channels. Significant contextual heterogeneity in the public reliance on information sources provides insight here. Hong Kong reported greater reliance on social media and far less trust in official websites, suggesting that official messaging in Hong Kong did not likely drive individual behavior change; by contrast, the UK results suggested that although the UK government possessed an effective platform to influence public health behavior, government health messaging was insufficient to attain similar baseline knowledge levels to those in Hong Kong, particularly in the absence of prior population sensitization to infectious disease outbreaks. Therefore, there is a pressing need to tailor communication approaches, likely on a graduated scale, but at a minimum in a binary fashion to accommodate both “naive” and “experienced” populations.

Third, the comparative snapshots of initial community responses captured by this study demonstrate the diversity in approaches and pandemic responses during the early phases of the COVID-19 pandemic. Across many contexts, national lockdowns became commonplace as the true magnitude of transmission became apparent; however, the associated indirect costs render blanket strategies untenable in the medium term. As national lockdowns are lifted, countries worldwide face the challenge of resurging cases and must consider nuanced approaches to prevent additional harm. Driven by anxiety, high perceived severity and knowledge, Hong Kong conducted widespread preventive measures early and en masse. Together with early government actions [33], the strategies adopted by the Hong Kong community were successful during the initial phase of the pandemic. Considering this—and that national populations are now highly sensitized to COVID-19 transmission—tailored public health messaging, early regional containment, and increased health capacity should ensure more effective public health responses with less indirect impact on national economies.

### Study Strengths and Limitations

From a methodological perspective, the UK sampling approach enabled the sample size to be achieved quickly, thereby accurately capturing prevailing sentiment and behavior across a short time frame (2 days). However, this approach likely came at the expense of excluding participants without access to the internet, and it contrasted with the survey period in Hong Kong (3 weeks); this likely led to some sampling bias, especially during the initial phase of the pandemic (when there was much uncertainty about the disease). Additionally, both samples varied across the demographic spectrum; thus, although responses were weighted, caution should be taken when extrapolating study findings to wider populations. Moreover, given the incompatibility of region-specific weights and the controversy in estimating standard errors when survey weights are involved [34], unweighted regression results were presented; however, they should be interpreted with caution. Last but not least, although both surveys were conducted early locally, the difference in surrounding international events during the survey periods (eg, the Hong Kong survey was conducted before COVID-19 was formally declared a pandemic, but the UK survey was launched after this declaration) may have introduced bias in the survey responses.

### Conclusions

This study compared the initial community responses to COVID-19 in Hong Kong and the United Kingdom. In line with the high baseline level of risk perception and knowledge and with historical exposure to respiratory disease outbreaks, the adoption of preventive measures was higher in Hong Kong. However, the UK sample demonstrated that this adoption could be improved by heightened risk perception and knowledge, best driven by improved public health campaigns. Together, these results suggest that health officials should ascertain baseline levels of risk perception and knowledge, as well as prior sensitization to infectious disease outbreaks, when developing mitigation strategies. Risk communication should be performed through suitable media channels—and trust should be maintained—while early intervention remains the cornerstone of effective outbreak response.

## Appendix

Multimedia Appendix 1 Supplementary files.

## Abbreviations

aOR: adjusted odds ratio
HASD-A: Hospital Anxiety and Depression scale–Anxiety
KAP: knowledge, attitudes, and practices

## References

[1] Rolling updates on coronavirus disease (COVID-19). World Health Organization.

[2] Countries/areas with reported cases of Coronavirus Disease 2019 (COVID-19) (Last updated on August 2, 2020, 11 am) 2020. Hong Kong Centre for Health Protection.

[3] Kwok KO, Lai FYL, Wei VWI, Tsoi MTF, Wong SYS, Tang JWT (2020). Comparing the impact of various interventions to control the spread of COVID-19 in twelve countries. J Hosp Infect.

[4] (2020). Prime Minister's statement on coronavirus (COVID-19): 16 March 2020. Prime Minister's Office of the Government of the United Kingdom.

[5] Kwok KO, Li KK, Chan HHH, Yi YY, Tang A, Wei WI, Wong SYS (2020). Community responses during early phase of COVID-19 epidemic, Hong Kong. Emerg Infect Dis.

[6] Leung GM, Ho L, Chan SKK, Ho S, Bacon-Shone J, Choy RYL, Hedley AJ, Lam T, Fielding R (2005). Longitudinal assessment of community psychobehavioral responses during and after the 2003 outbreak of severe acute respiratory syndrome in Hong Kong. Clin Infect Dis.

[7] Bish A, Michie S (2010). Demographic and attitudinal determinants of protective behaviours during a pandemic: a review. Br J Health Psychol.

[8] Jones JH, Salathé M (2009). Early assessment of anxiety and behavioral response to novel swine-origin influenza A (H1N1). PLoS One.

[9] Bults M, Beaujean DJ, de Zwart O, Kok G, van Empelen P, van Steenbergen JE, Richardus JH, Voeten HA (2011). Perceived risk, anxiety, and behavioural responses of the general public during the early phase of the Influenza A (H1N1) pandemic in the Netherlands: results of three consecutive online surveys. BMC Public Health.

[10] Lohiniva A, Sane J, Sibenberg K, Puumalainen T, Salminen M (2020). Understanding coronavirus disease (COVID-19) risk perceptions among the public to enhance risk communication efforts: a practical approach for outbreaks, Finland, February 2020. Euro Surveill.

[11] Gesser-Edelsburg A, Cohen R, Hijazi R, Abed Elhadi Shahbari N (2020). Analysis of public perception of the Israeli government's early emergency instructions regarding COVID-19: online survey study. J Med Internet Res.

[12] Motta Zanin G, Gentile E, Parisi A, Spasiano D (2020). A preliminary evaluation of the public risk perception related to the COVID-19 health emergency in Italy. Int J Environ Res Public Health.

[13] Olapegba PO, Iorfa SK, Kolawole SQ, Oguntayo Rotimi, Gandi Joshua C, Ottu Iboro F A, Ayandele Olusola (2020). Survey data of COVID-19-related knowledge, risk perceptions and precautionary behavior among Nigerians. Data Brief.

[14] McFadden SM, Malik AA, Aguolu OG, Willebrand KS, Omer SB (2020). Perceptions of the adult US population regarding the novel coronavirus outbreak. PLoS One.

[15] Clements JM (2020). Knowledge and behaviors toward COVID-19 among US residents during the early days of the pandemic: cross-sectional online questionnaire. JMIR Public Health Surveill.

[16] Husnayain A, Shim E, Fuad A, Su EC (2020). Understanding the community risk perceptions of the COVID-19 outbreak in South Korea: infodemiology study. J Med Internet Res.

[17] Huynh TLD (2020). Data for understanding the risk perception of COVID-19 from Vietnamese sample. Data Brief.

[18] Christina JA, Leigh B, Charlotte V, Rozlyn R, Philippa P, Jeffrey WE, Helen W Perceptions and behavioural responses of the general public during the COVID-19 pandemic: A cross-sectional survey of UK Adults. medRxiv (forthcoming).

[19] Latest situation of cases of COVID-19. Hong Kong Centre for Health Protection.

[20] Coronavirus (COVID-19) in the UK: Cases in United Kingdom. Government of the United Kingdom.

[21] ESOMAR 28: 28 questions to help online research buyers. YouGov.

[22] COVID 19 - population survey tool. The Chinese University of Hong Kong.

[23] COVID-19 scientific resources. MRC Centre for Global Infectious Disease Analysis, Imperial College London.

[24] Snaith RP (2003). The Hospital Anxiety And Depression Scale. Health Qual Life Outcomes.

[25] 2011 Census. UK Office for National Statistics.

[26] By-census results 2016. Hong Kong Census and Statistics Department.

[27] Anderson L, Fricker RD (2015). Raking: an important and often overlooked survey analysis tool. Phalanx.

[28] Rabie T, Curtis V (2006). Handwashing and risk of respiratory infections: a quantitative systematic review. Trop Med Int Health.

[29] SteelFisher GK, Blendon RJ, Ward JRM, Rapoport R, Kahn EB, Kohl KS (2012). Public response to the 2009 influenza A H1N1 pandemic: a polling study in five countries. Lancet Infect Dis.

[30] Funk S, Salathé M, Jansen VAA (2010). Modelling the influence of human behaviour on the spread of infectious diseases: a review. J R Soc Interface.

[31] de Zwart O, Veldhuijzen IK, Elam G, Aro AR, Abraham T, Bishop GD, Voeten HACM, Richardus JH, Brug J (2009). Perceived threat, risk perception, and efficacy beliefs related to SARS and other (emerging) infectious diseases: results of an international survey. Int J Behav Med.

[32] Bults M, Beaujean DJMA, Richardus JH, Voeten HACM (2015). Perceptions and behavioral responses of the general public during the 2009 influenza A (H1N1) pandemic: a systematic review. Disaster Med Public Health Prep.

[33] Wong SYS, Kwok KO, Chan FKL (2020). What can countries learn from Hong Kong's response to the COVID-19 pandemic?. CMAJ.

[34] Gelman A (2007). Struggles with survey weighting and regression modeling. Statist Sci.

