# Supplementary material for: Comparing Public Perceptions and Preventive Behaviors During the Early Phase of the COVID-19 Pandemic in Hong Kong and the United Kingdom: Cross-sectional Survey Study
Source: J Med Internet Res. 2021 Mar 8;23(3):e23231. doi: 10.2196/23231 (PMC7942393; doi:10.2196/23231)
Supplement: Multimedia Appendix 1 [file jmir_v23i3e23231_app1.docx]

**SUPPLEMENTARY INFORMATION**

Table S1. Access and perceived reliability of information sources

| **Information sources** |  | **Access** | | | | | | |  | **Perceived reliability** | | | | | | |
| --- | --- | --- | --- | --- | --- | --- | --- | --- | --- | --- | --- | --- | --- | --- | --- | --- |
|  |  | **UK (n=1768)** | | |  | **HK (n=1663)** | | |  | **UK (n=1768)** | | |  | **HK (n=1663)** | | |
|  |  | **N** | **% (unweighted)** | **% (weighted)** |  | **N** | **% (unweighted)** | **% (weighted)** |  | **N** | **% (unweighted)** | **% (weighted)** |  | **N** | **% (unweighted)** | **% (weighted)** |
| **Public channel** |  |  |  |  |  |  |  |  |  |  |  |  |  |  |  |  |
| Social media platform |  | 625 | 35.4 | 36.0 |  | 1560 | 93.8 | 92.1 |  | 187 | 10.6 | 10.9 |  | 434 | 26.1 | 27.8 |
| Newspapers |  | 827 | 46.8 | 46.3 |  | 665 | 40.0 | 38.7 |  | - | - | - |  | 907 | 54.5 | 51.2 |
| Magazines |  | 58 | 3.3 | 3.3 |  | 104 | 6.3 | 7.4 |  | - | - | - |  | 364 | 21.9 | 20.4 |
| Radio |  | 721 | 40.8 | 40.7 |  | 569 | 34.2 | 36.3 |  | - | - | - |  | 959 | 57.7 | 55.4 |
| Television |  | 1305 | 73.8 | 73.5^a^ |  | 1155 | 69.5 | 68.1 |  | - | - | - |  | 651 | 39.1 | 38.0 |
| Streaming |  |  |  |  |  |  |  |  |  | 139 | 7.9 | 8.1 |  | - | - | - |
| Official websites |  | 1126 | 63.7 | 64.0^b^ |  | 1496 | 90.0 | 89.3 |  | 1602 | 90.6 | 90.7 |  | 260 | 15.6 | 15.6 |
| Unofficial websites |  |  |  |  |  |  |  |  |  | 138 | 7.8 | 8.1 |  | 380 | 22.9 | 26.4 |
| **Personal communication** |  |  |  |  |  |  |  |  |  |  |  |  |  |  |  |  |
| My families/friends |  | 866 | 49.0 | 49.4^c^ |  | 931 | 56.0 | 53.9 |  | 582 | 32.9 | 33.0 |  | 593 | 35.7 | 40.6 |
| Work/school/college |  |  |  |  |  |  |  |  |  | 828 | 46.8 | 47.5 |  | - | - | - |
| **Professionals** |  |  |  |  |  |  |  |  |  |  |  |  |  |  |  |  |
| My doctor |  | 202 | 11.4 | 11.5^d^ |  | 86 | 5.2 | 4.8 |  | 1542 | 87.2 | 87.2 |  | 1408 | 84.7 | 84.3 |
| Other health professionals |  |  |  |  |  |  |  |  |  | 1497 | 84.7 | 84.7 |  | - | - | - |
| **Others** |  |  |  |  |  |  |  |  |  |  |  |  |  |  |  |  |
| Others |  | 9 | 0.5 | 0.5 |  | 10 | 0.6 | 0.7 |  | - | - | - |  | - | - | - |
| ^a^ UK separately asked about the two options, with the number of respondents reporting access to television being 1292 (unweighted:73.1%; weighted 72.7%) and that to streaming services being 31 (unweighted: 1.8%; weighted:1.8%). | | | | | | | | | | | | | | | | |
| ^b^ UK separately asked about the two options, with the number of respondents reporting access to official websites being 1101 (unweighted:62.3%; weighted:62.6%) and that to unofficial websites being 166 (unweighted:9.4%; weighted:9.8%). | | | | | | | | | | | | | | | | |
| ^c^ UK separately asked about the two options, with the number of respondents reporting access to my families/friends being 577 (unweighted: 32.6%; weighted: 32.8%) and that to work/school/college being 537 (unweighted: 30.4%; weighted: 31.1%). | | | | | | | | | | | | | | | | |
| ^d^ UK separately asked about the two options, with the number of respondents reporting access to my doctor being 82 (unweighted: 4.6%; weighted: 4.6%) and that to other health professionals being 140 (unweighted: 7.9%; weighted: 8.0%). | | | | | | | | | | | | | | | | |

Table S2. Proportion of respondents adopting precautionary measures against COVID-19

| **Precautionary measures** |  | **Proportion of respondents** | | | | |
| --- | --- | --- | --- | --- | --- | --- |
|  |  | **UK** | |  | **HK** | |
|  |  | **% (unweighted)** | **% (weighted)** |  | **% (unweighted)** | **% (weighted)** |
| **Travel restriction (avoid going to…)** |  |  |  |  |  |  |
| Affected areas^a^ in the world |  | 35.2 | 34.6 |  | 92.4 | 92.3 |
| **Social distancing (avoid…)** |  |  |  |  |  |  |
| **General** |  |  |  |  |  |  |
| Crowded areas |  | 59.6 | 59.0 |  | 88.2 | 87.2 |
| Social events |  | 57.2 | 56.6 |  | 64.3 | 63.1 |
| Going out |  | 37.9 | 37.8 |  | 70.1 | 68.0 |
| **Contact** |  |  |  |  |  |  |
| Contacting people who have a fever or respiratory symptoms |  | 50.6 | 50.1 |  | 93.2 | 93.7 |
| Contacting people who have been to specific regions^b^ in a limited period^b^ |  | 34.0 | 33.7 |  | 84.8 | 83.8 |
| **Work** |  |  |  |  |  |  |
| Going to work^c^ |  | 22.5 | 22.5 |  | 35.4 | 32.6 |
| **Others** |  |  |  |  |  |  |
| Taking public transportation |  | 40.9 | 40.2 |  | 39.4 | 40.0 |
| Going to hospitals or other healthcare settings |  | 31.1 | 30.9 |  | 76.2 | 75.5 |
| Going to wet market |  | 21.0 | 20.6 |  | 69.4 | 67.2 |
| Going to school/ letting your children go to school ^d^ |  | 17.0 | 17.6 |  | 50.9 | 50.1 |
| **Personal hygiene** |  |  |  |  |  |  |
| Clean hands |  | 91.5 | 91.3 |  | 95.9 | 95.0 |
| Cover mouth and nose when sneezing or coughing |  | 74.3 | 74.2 |  | 97.2 | 97.1 |
| Disinfect home |  | 26.6 | 26.6 |  | 78.2 | 77.9 |
| Wear mask |  | 3.0 | 3.1 |  | 99.0 | 98.8 |
| ^a^ Affected areas refer to China (HK) / affected areas in the world (UK). | | | | | | |
| ^b^ "Specific regions in a limited period" refers to affected areas (UK) / Wuhan (HK) in the past 14 days (UK) / past one month (HK). | | | | | | |
| ^c^ Only included respondents who were employees or employers (n=2160). | | | | | | |
| ^d^ Only included respondents who were full-time students or had at least one child (n=1239). | | | | | | |

Table S3. Univariate analysis of factors associated with the adoption of different types of social distancing

| **Factors** |  | **Types of social distancing** | | | | | | | |  |
| --- | --- | --- | --- | --- | --- | --- | --- | --- | --- | --- |
|  |  | **General ^a^ (n=3431)** | |  | **Contact ^b^ (n=3431)** | |  | **Work ^c^ (n=2160)** | | |
|  |  | **OR (95% CI)** | ***P*-value** |  | **OR (95% CI)** | ***P*-value** |  | **OR (95% CI)** | ***P*-value** | |
| **Age** |  |  |  |  |  |  |  |  |  | |
| 18-24 |  | - | - |  | - | - |  | - | - | |
| 25-34 |  | 1.30 (1.05, 1.60) | .02 |  | 0.87 (0.69, 1.09) | .23 |  | 1.04 (0.76, 1.44) | .80 | |
| 35-44 |  | 0.91 (0.73, 1.13) | .40 |  | 0.50 (0.40, 0.63) | <.001 |  | 0.88 (0.63, 1.22) | .43 | |
| 45-54 |  | 0.81 (0.64, 1.04) | .09 |  | 0.35 (0.28, 0.46) | <.001 |  | 0.56 (0.39, 0.81) | .002 | |
| 55+ |  | 0.64 (0.52, 0.79) | <.001 |  | 0.28 (0.22, 0.34) | <.001 |  | 0.43 (0.28, 0.65) | <.001 | |
| **Sex** |  |  |  |  |  |  |  |  |  | |
| Female |  | - | - |  | - | - |  | - | - | |
| Male |  | 0.68 (0.59, 0.78) | <.001 |  | 0.56 (0.49, 0.64) | <.001 |  | 0.86 (0.71, 1.04) | .11 | |
| **Education attainment ^d^** |  |  |  |  |  |  |  |  |  | |
| No formal qualification/  lower secondary or below |  | - | - |  | - | - |  | - | - | |
| Secondary level qualification/  higher secondary |  | 0.82 (0.58, 1.17) | .27 |  | 0.80 (0.57, 1.12) | .19 |  | 1.03 (0.47, 2.30) | .94 | |
| Post-secondary but below degree |  | 1.03 (0.72, 1.48) | .87 |  | 1.26 (0.88, 1.79) | .21 |  | 1.25 (0.56, 2.80) | .58 | |
| Degree or above |  | 1.43 (1.02, 2.01) | .04 |  | 1.75 (1.25, 2.44) | <.001 |  | 2.48 (1.14, 5.38) | .02 | |
| **Employment status** |  |  |  |  |  |  |  |  |  | |
| Employed |  | - | - |  | - | - |  | - | - | |
| Full-time student |  | 1.32 (1.06, 1.65) | .01 |  | 2.08 (1.63, 2.65) | <.001 |  | - | - | |
| Unemployed |  | 1.51 (1.21, 1.87) | <.001 |  | 1.19 (0.95, 1.48) | .13 |  | - | - | |
| Retired |  | 0.85 (0.70, 1.04) | .11 |  | 0.49 (0.41, 0.60) | <.001 |  | - | - | |
| **Setting** |  |  |  |  |  |  |  |  |  | |
| HK |  | - | - |  | - | - |  | - | - | |
| UK |  | 0.34 (0.29, 0.39) | <.001 |  | 0.08 (0.07, 0.10) | <.001 |  | 0.53 (0.44, 0.64) | <.001 | |
| **Perceived severity** |  |  |  |  |  |  |  |  |  | |
| Not serious |  | - | - |  | - | - |  | - | - | |
| Serious |  | 3.54 (3.05, 4.11) | <.001 |  | 8.19 (7.02, 9.56) | <.001 |  | 1.82 (1.50, 2.21) | <.001 | |
| **Perceived ease of transmission** |  |  |  |  |  |  |  |  |  | |
| Difficult |  | - | - |  | - | - |  | - | - | |
| Easy |  | 2.52 (2.08, 3.06) | <.001 |  | 3.51 (2.92, 4.23) | <.001 |  | 1.42 (1.10, 1.84) | 0.01 | |
| **Anxiety level** |  |  |  |  |  |  |  |  |  | |
| Normal |  | - | - |  | - | - |  | - | - | |
| Borderline abnormal |  | 1.84 (1.55, 2.18) | <.001 |  | 1.97 (1.66, 2.33) | <.001 |  | 1.64 (1.30, 2.08) | <.001 | |
| Abnormal |  | 2.43 (2.06, 2.85) | <.001 |  | 2.14 (1.82, 2.52) | <.001 |  | 1.82 (1.46, 2.26) | <.001 | |
| ^a^ "General" refers to avoiding going to: (i) crowded areas; (ii) social events; and (iii) going out. | | | | | | | | | |  |
| ^b^ "Contact " refers to avoiding contacting individuals who (i) had a fever or respiratory symptoms; and (ii) had been to affected areas (UK) / Wuhan (HK) in the past 14 days (UK) / past month (HK). | | | | | | | | | |  |
| ^c^ “Work” refers to avoiding going to work. | | | | | | | | | |  |
